# Safety and effectiveness of mycophenolate mofetil associated with tacrolimus for liver transplantation immunosuppression: a systematic review and meta-analysis of randomized controlled trials

**DOI:** 10.6061/clinics/2021/e2597

**Published:** 2021-03-01

**Authors:** Francisco Tustumi, Antonio Afonso de Miranda, Sérgio Silveira, Felipe Alexandre Fernandes, Miller Barreto de Brito e Silva, Lucas Ernani, Lucas Souto Nacif, Fabricio Ferreira Coelho, Wellington Andraus, Wanderley Marques Bernardo, Paulo Herman, Luiz Augusto Carneiro-D’Albuquerque

**Affiliations:** Departamento de Gastroenterologia, Hospital das Clinicas HCFMUSP, Faculdade de Medicina, Universidade de Sao Paulo, Sao Paulo, SP, BR

**Keywords:** Mycophenolic Acid, Tacrolimus, Transplantation, Immunosuppressive Agents

## Abstract

A combination of immunosuppressants may improve outcomes due to the synergistic effect of their different action mechanisms. Currently, there is no consensus regarding the best immunosuppressive protocol after liver transplantation. This review aimed to evaluate the effectiveness and safety of tacrolimus associated with mycophenolate mofetil (MMF) in patients undergoing liver transplantation. We performed a systematic review and meta-analysis of randomized clinical trials. Eight randomized trials were included. The proportion of patients with at least one adverse event related to the immunosuppression scheme with tacrolimus associated with MMF was 39.9%. The tacrolimus with MMF immunosuppression regimen was superior in preventing acute cellular rejection compared with that of tacrolimus alone (risk difference [RD]=-0.11; *p* =0.001). The tacrolimus plus MMF regimen showed no difference in the risk of adverse events compared to that of tacrolimus alone (RD=0.7; *p*=0.66) and cyclosporine plus MMF (RD=-0.7; *p*=0.37). Patients undergoing liver transplantation who received tacrolimus plus MMF had similar adverse events when compared to patients receiving other evaluated immunosuppressive regimens and had a lower risk of acute rejection than those receiving in the monodrug tacrolimus regimen.

## INTRODUCTION

Immunosuppression protocols improve survival and decrease acute rejection episodes in patients who undergo liver transplantation (1). Over the past few decades, several immunosuppressive therapy schemes have been developed, and the crucial choice of the regimen scheme is related to its effectiveness and safety. The ideal strategy would provide a better immunosuppressive effect, with low rejection rates and few side effects (1).

Tacrolimus (FK506) has shown excellent effectiveness in immunosuppression for solid organ transplantation. However, its common side effects require constant monitoring of the drug’s serum level. The most commonly reported side effects are renal failure, neurotoxicity, changes in blood glucose, and susceptibility to infections or neoplasms (2).

Mycophenolate mofetil (MMF) has been shown to decrease the risk of acute and late rejections in immunosuppression protocols (3). MMF has been used to reinforce the action of FK506, potentially reducing the required dose of tacrolimus and thus its side effects. Moreover, this combination could reduce the need for corticosteroids, whose long-term side effects, such as diabetes, hypertension, and hypercholesterolemia, are deleterious (4). A combination of immunosuppressants may improve the outcomes due to the synergistic effect based on their different action mechanisms (5). A combination of immunosuppressants aims to obtain the maximum effectiveness in preventing rejection and mortality, along with minimal adverse events.

Currently, consensus regarding the best immunosuppressive protocol after liver transplantation is still lacking. This review aimed to evaluate the efficiency and safety of MMF associated with tacrolimus in patients undergoing liver transplantation.

## METHODS

The Institutional Ethics Committee approved this study protocol. This study was conducted following the PRISMA statement (6). The research protocol was registered in the International Prospective Register of Systematic Reviews (http://www.crd.york.ac.uk/ PROSPERO; with the number CRD42020195950).

### Database search

A systematic review was performed in PubMed, EMBASE, Cochrane Library Central, SciELO/LILACS, and gray literature searches for randomized controlled trials (RCTs) that compared immunosuppressant regimens combining tacrolimus and MMF with other regimens in patients who underwent liver transplantation. No restrictions were set for language or period. Furthermore, the references of the retrieved articles were cross-checked manually for additional studies. Only the publications with the most complete data were included when more than one study from a single center was found. The last search was conducted in June 2020.

Literature searches were performed in PubMed as follows: ((“mycophenolic acid“[MeSH Terms] OR (“mycophenolic“[All Fields] AND “acid“[All Fields]) OR “mycophenolic acid“[All Fields] OR “cellcept“[All Fields]) OR (“mycophenolic acid“[MeSH Terms] OR (“mycophenolic“[All Fields] AND “acid“[All Fields]) OR “mycophenolic acid“[All Fields] OR (“mycophenolate“[All Fields] AND “mofetil“[All Fields]) OR “mycophenolate mofetil“[All Fields])) AND ((((“tacrolimus“[MeSH Terms] OR “tacrolimus“[All Fields]) OR (“tacrolimus“[MeSH Terms] OR “tacrolimus“[All Fields])) OR kujimycin[All Fields]) OR (“tacrolimus“[MeSH Terms] OR “tacrolimus“[All Fields] OR “fk506“[All Fields])) AND Clinical Trial[ptyp]. For EMBASE, Cochrane Library Central, and SciELO/LILACS, the search was performed with the same medical subject headings (MeSH) and keywords in various combinations.

### Study selection

Two reviewers, using predefined inclusion and exclusion criteria, performed independent eligibility assessments to select the studies. Any disagreement on the inclusion or exclusion of a given study was resolved by consensus. The inclusion criteria were (I) adult patients submitted to liver transplantation and (II) RCTs that included immunosuppressive regimens with a combination of MMF and FK506 in one comparison arm irrespective of the number of patents. The exclusion criteria were: (I) reviews, case reports, editorials, letters, conference proceedings, and observational studies, (II) animal models, (III) studies from which the necessary data could not be extracted from the pooled results, and (IV) studies with no full text.

### Outcomes

The outcomes evaluated were the frequency of acute cellular rejection, graft loss, adverse event rate, and mortality rate during immunosuppressive regimen follow-up.

### Assessment of study quality

Study quality was assessed using Robins II (7), and certainty assessment was performed using GRADE (8).

### Statistical analysis

The absolute numbers for the outcome parameters were extracted and analyzed with Comprehensive Meta-Analysis software, version 2 (Engelwood, NJ; Biostat, 2014) for estimating the rates in one group and Review Manager Version 5.4 software (Copenhagen, The Nordic Cochrane Centre; the Cochrane Collaboration, 2014) for the comparison of the two groups. Fixed- and random-effect models were employed according to the level of heterogeneity. The summary weighted risk difference (RD) and the 95% confidence interval (95% IC) were calculated using the Mantel Haenszel test for categorical variables. The meta-analysis results were expressed through forest plots, and a funnel plot was used to identify publication bias.

## RESULTS

The literature search yielded 6,825 potentially relevant articles. After applying the inclusion/exclusion criteria, 8 RCTs (9-16) were included in the meta-analysis (Supplementary File 1). Table 1 describes the baseline characteristics of the included studies. The FK506 plus MMF regimen was compared with the control groups, including FK506 in isolation (6 articles (9,11-14,16)) and cyclosporine (CyA) associated with MMF (2 articles (10,15)). The mean follow-up time for the included patients was 27.7±19 months. Robins II is reported in Supplementary File 2 and GRADE in Supplementary Files 3 and 4. Funnel plots are shown in Supplementary Files 5 and 6.

### Adverse events of the FK506 plus MMF regimen

**Figure 1:**
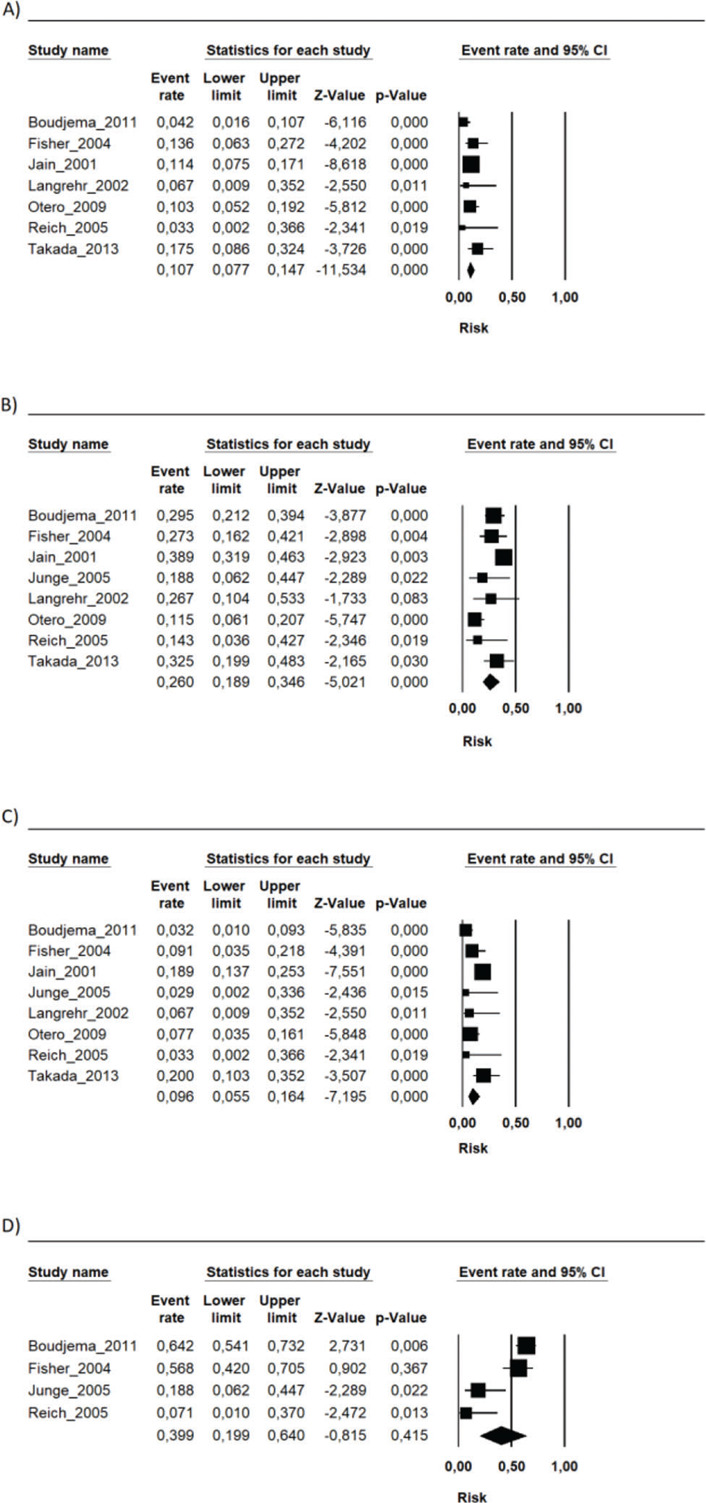
The risks of the FK506 plus MMF regimen on A) Graft loss; B) Acute rejection; C) Mortality; D) Adverse events.

**Figure 2:**
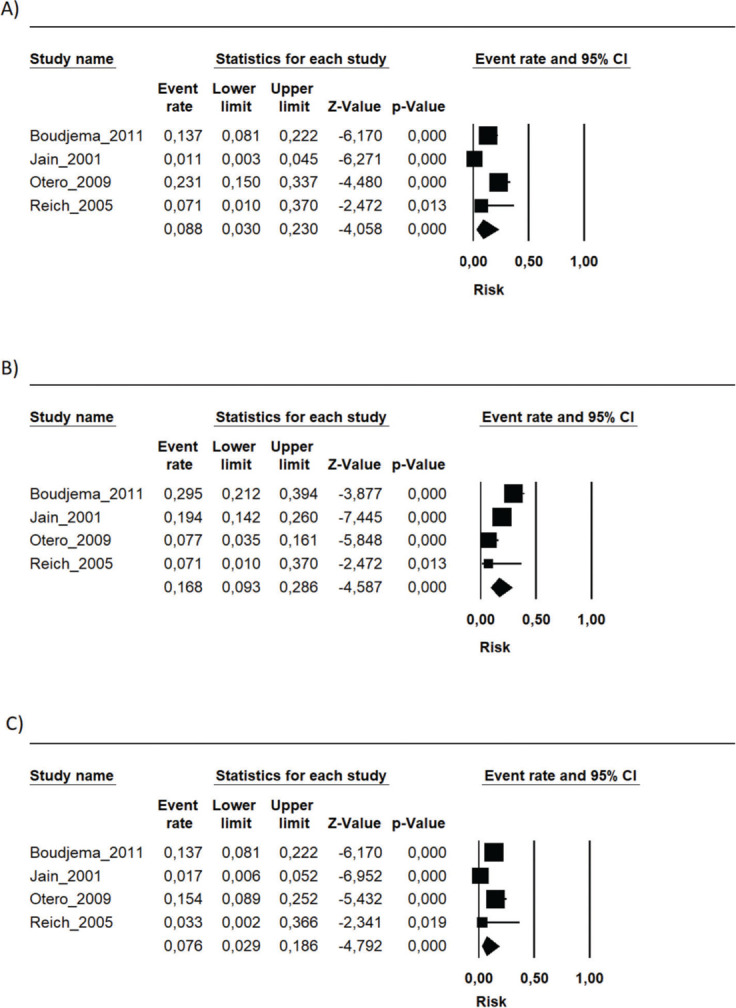
The main hematological adverse events related to the FK506 plus MMF regimen usage. A) Anemia; B) Leukopenia; C) Thrombocytopenia.

**Figure 3:**
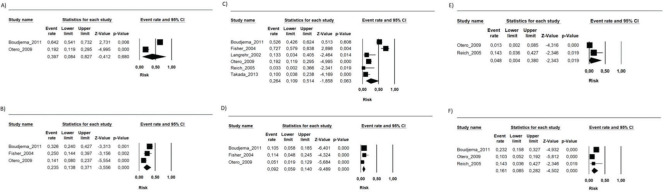
The risks of the FK506 plus MMF regimen on A) Acute kidney injury; B) New onset diabetes; C) Infections; D) CMV infection; E) Nausea/Vomiting; F) Diarrhea.

During the follow-up period in each study, the risk of mortality (related or not to the use of the immunosuppressive regimen) in the transplant recipients who used the FK506 associated with MMF regimen was 9.6% (95% CI 5.5-16.4%) (9-16). The proportion of patients with at least one adverse event related to the immunosuppression scheme was 39.9% (95% CI 19.9-64%) (9,10,12,15). The most frequently reported adverse events were gastrointestinal symptoms (nausea, vomiting, and diarrhea), infections, renal insufficiency, and hematology changes (leukopenia, anemia, and thrombocytopenia) (9-16) (Figures 1, 2 and 3 and Table 2).

### MMF associated with FK506 *vs*. FK506 in isolation

**Figure 4:**
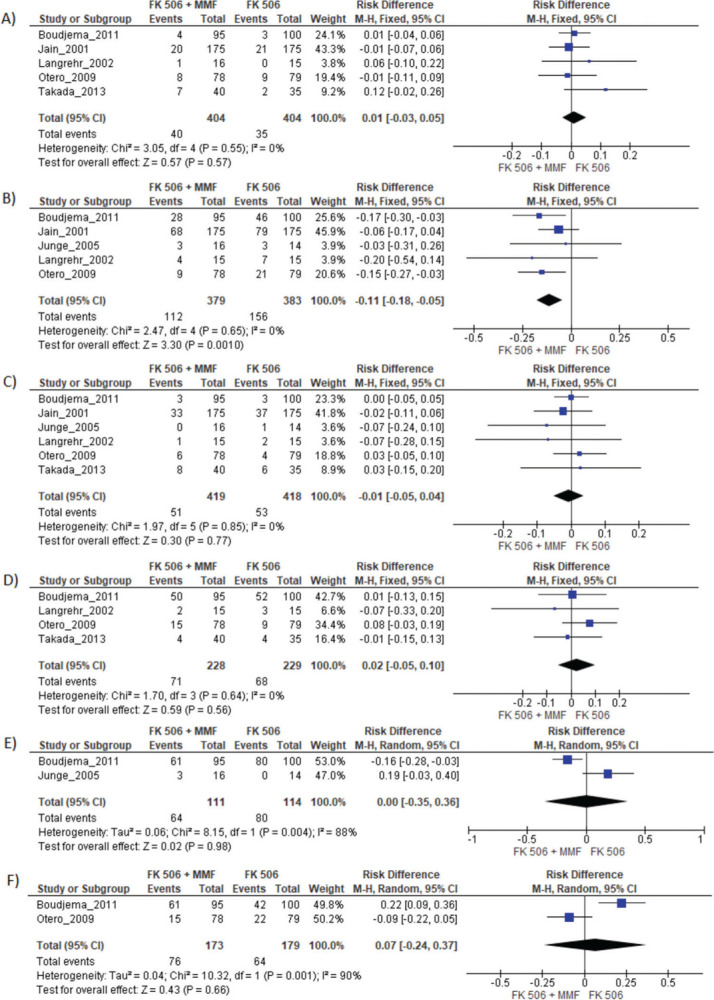
FK506 plus MMF compared with FK506 in isolation. A) Graft loss; B) Acute rejection; C) Mortality; D) Infection; E) Adverse events; F) Acute kidney injury.

MMF associated with the FK506 regimen was compared with FK506 in isolation (9,11-14,16) (Table 3 and Figure 4).

Regarding the effectiveness, FK506 with the MMF immunosuppression regimen was superior in preventing acute rejection when compared with FK506 in isolation (RD=-0.11; 95% CI: -0.18 to -0.05; I^2^=0; *p*=0.001; fixed-effects model; certainty assessment: moderate) (9,11,13,14,16). There was no difference regarding the risk of graft loss among the immunosuppression regimens evaluated (RD=0.01; 95% CI: -0.03 to 0.05; I^2^=0; *p*=0.57; fixed-effects model; certainty assessment: moderate) (9,11,13,14,16).

Regarding safety, there was no difference in the risk of death during follow-up among the immunosuppression regimens (RD=-0.01; 95% CI: -0.05 to 0.04; I^2^=0%; *p*=0.77; fixed-effects model; certainty assessment: moderate) (9,11-14,16). Similarly, no difference in the risk of renal failure (RD=0.07; 95% CI: -0.24 to 0.37; I^2^=90%; *p*=0.98; random-effects model; certainty assessment: very low) (9,14) or the risk of infections (RD=0.02; 95% CI: -0.05 to 0.10; I2=0%; *p*=0.56; fixed-effects model; certainty assessment: moderate) was found (9,13,14,16). There was no significant difference in the proportion of patients who had at least one adverse event related to the immunosuppressant (RD=0.00; 95% CI: -0.35 to 0.36; I^2^=88%; *p*=0.66; random-effects model; certainty assessment: very low) (9,12).

### MMF plus FK506 *vs*. CyA plus MMF

**Figure 5:**
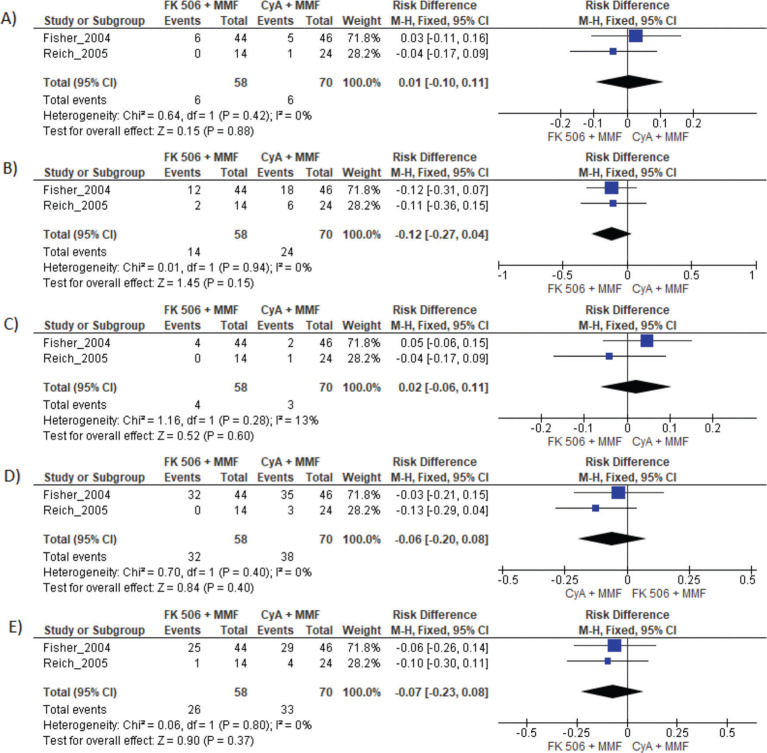
FK506 plus MMF compared with CyA plus MMF. A) Graft loss; B) Acute rejection; C) Mortality; D) Infection; E) Adverse events.

Two studies compared these schemes (10,15). No difference in graft loss, rejection, mortality, infections, or the proportion of patients who had at least one adverse event was found (Table 3 and Figure 5).

## DISCUSSION

The results of the present systematic review and meta-analysis of randomized clinical trials showed that liver transplantation patients using MMF plus FK506 had a lower risk for acute rejection than those using isolated FK506. The FK506 plus MMF regimen showed a high risk of adverse events. Almost 40% of the patients suffered at least one adverse event during the follow-up. However, compared to FK506 in isolation or CyA plus MMF, the risk for adverse events was similar.

FK506 reduces CD4 and CD8 T-cell proliferation (17). MMF inhibits T-lymphocyte proliferation by limiting DNA synthesis in these cells and increasing their apoptosis (18). Additionally, MMF and FK506 (in association or with other immunosuppressive agents) inhibit the proliferation of human B lymphocytes and immunoglobulin expression (19).

Calcineurin inhibitors such as CyA and FK506, due to their tubulointerstitial, glomerular, vascular, and microangiopathic effects, increase the risk for acute or chronic nephrotoxicity (20). One could expect that adding MMF to FK506 would reduce the risk of kidney injury. However, in the present study, the cumulative risk for acute kidney injury was not different from that of the other immunosuppressive regimens. Additionally, in this review, two studies (11,16) were not submitted to the quantitative analysis when comparing FK506 in isolation with FK506 plus MMF, given the unavailability of vital data for analysis. These studies also showed that there was no difference in the mean serum creatinine and urea. Thus, the risk for acute kidney injury of FK506 was not reduced when MMF was added to the scheme. Nonetheless, we found elevated heterogeneity for the outcome “acute kidney injury”, and in fact, the overall certainty of the evidence was classified as very low for this outcome.

It is known that CyA nephrotoxicity is slightly higher than FK506 nephrotoxicity; however, none of the studies evaluated the difference in the risk for acute kidney injury between the regimens FK506 plus MMF and CyA plus MMF (20).

In this study, the graft loss rates were similar across all the immunosuppressive regimens. However, the use of FK506 plus MMF was associated with a lower risk for acute rejection than isolated FK506 (RD=-0.11; 95% CI: -0.18 to -0.05; *p*=0.001), with a moderate certainty assessment. The use of a combination of immunosuppressive drugs, instead of FK506 in isolation, has the potential for synergic action, as FK506 and MMF act by different mechanisms (5). Tacrolimus is a macrolide produced by the fungus *Streptomyces tsukubaensis* that has calcineurin inhibitor properties (21). Mycophenolate mofetil is a potent, selective, and reversible inhibitor of inosine monophosphate dehydrogenase (3).

As strength of the present study, only randomized clinical trials were included. Thus, the risk of bias is mitigated but the results may be affected by performance and detection bias due to the lack of blinding in the included studies (Supplementary File 2). The quality of evidence evaluated by the GRADE tool showed a moderate certainty of evidence for most of the outcomes. The main weak point in the quality of evidence was the small sample size in most of the included studies, leading to a high level of imprecision.

Another limitation of this review was the heterogeneous serum FK506 targets, which varied depending on the studies and the MMF dosage. Clinical heterogeneity due to differences in the populations, such as the baseline liver disease and distinct inductive approaches adopted by the trials may have impacted the heterogeneity found in the outcomes “adverse events” and “acute kidney injury” in the comparison of MMF plus FK506 *vs*. FK506 in isolation. Additionally, in the included studies, the sample size was small, and the mean follow-up varied. Also, some of the included studies were published years ago, which may have also influenced the results considering the improvements in liver transplantation care in recent years. Future well-designed RCTs with long-term follow-up are warranted.

## CONCLUSION

The use of MMF associated with FK506 in patients undergoing liver transplantation shows similar adverse events when compared to patients receiving other immunosuppressive regimens. Patients using this association seem to have a lower risk of acute rejection than those using FK506 alone.

## AUTHOR CONTRIBUTIONS

All authors contributed to this study and participated in the writing and drafting of the manuscript or critically revised it for relevant intellectual content. Tustumi F contributed to the conception or design of the work. Ernani L contributed to the acquisition of the data. Fernandes FA contributed to the analysis, or interpretation of data for the work. All authors have approved the final and submitted version to be published and assumed joint accountability for all aspects of the work in ensuring that questions related to the accuracy or integrity of any part of the work are appropriately investigated and resolved.

## Figures and Tables

**Table 1:** Baseline characteristics of the included studies.

Author	Year	Design	Follow-up (mo)	Intervention group			Control group	
				Tacrolimus target level	MMF (daily dosage)	N	Regimen	N
Boudjema et al. (9)	2011	RCT	12	6-12 ng/ml	2-3 g	95	FK506	100
Fisher et al. (10)	2004	RCT	48	5-15 ng/ml	1-3 g	44	CyA+MMF	46
Jain et al. (11)	2001	RCT	36	8-12 ng/ml	2 g	175	FK506	175
Junge et al. (12)	2005	RCT	24	5-8 ng/ml	1-2 g	16	FK506	14
Langrehr et al. (13)	2002	RCT	24	NR	NR	15	FK506	15
Otero et al. (14)	2009	RCT	6	5-15 ng/ml	2 g	78	FK506	79
Reich et al. (15)	2005	RCT	12	NR	3 g	14	CyA+MMF	24
Takada et al. (16)	2013	RCT	60	5-15 ng/ml	10-30 mg/kg	40	FK506	35

RCT: randomized clinical trial; mo: months; MMF: Mycophenolate mofetil; FK506: Tacrolimus; CyA: Cyclosporine; NR: not reported.

**Table 2:** Summary of the risk of occurrence of the main adverse events associated with the FK506 associated with MMF immunosuppression regimen.

Adverse event	Risk (%)	CI 95%
Anemia	8.8	3-23
Leukopenia	16.8	9.3-28.6
Thrombocytopenia	7.6	2.9-18.6
Infections	26.4	10.9-51.4
CMV infection	9.2	5.9-14
Acute kidney injury	39.7	8.4-82.7
Diabetes	23.5	13.8-37.1
Diarrhea	16.1	8.5-28.2
Nausea	4.8	4-38

**Table 3:** Results of FK 506 plus MMF regimen when compared FK 506 and CyA plus MMF. MMF: Mycophenolate mofetil; FK 506: Tacrolimus; CyA: Cyclosporine.

FK506	Risk difference	CI 95%	*p*-value
Graft loss	0.01	-0.03 to 0.05	*0.57*
Acute rejection	-0.11	-0.18 to -0.05	*0.001*
Mortality	-0.01	-0.01 to 0.04	*0.77*
Infection	0.02	-0.02 to 0.10	*0.56*
Adverse events	0.00	-0.35 to 0.36	*0.98*
Acute kidney injury	0.07	-0.24 to .37	*0.66*
**CyA and MMF**	**Risk difference**	**CI 95%**	***p*-value**
Graft loss	0.01	-0.10 to 0.11	*0.88*
Acute rejection	-0.12	-0.27 to 0.04	*0.94*
Mortality	0.02	-0.06 to 0.11	*0.28*
Infection	-0.06	-0.20 to 0.08	*0.40*
Adverse events	-0.07	-0.23 to 0.08	*0.37*

**Supplementary File 2:**
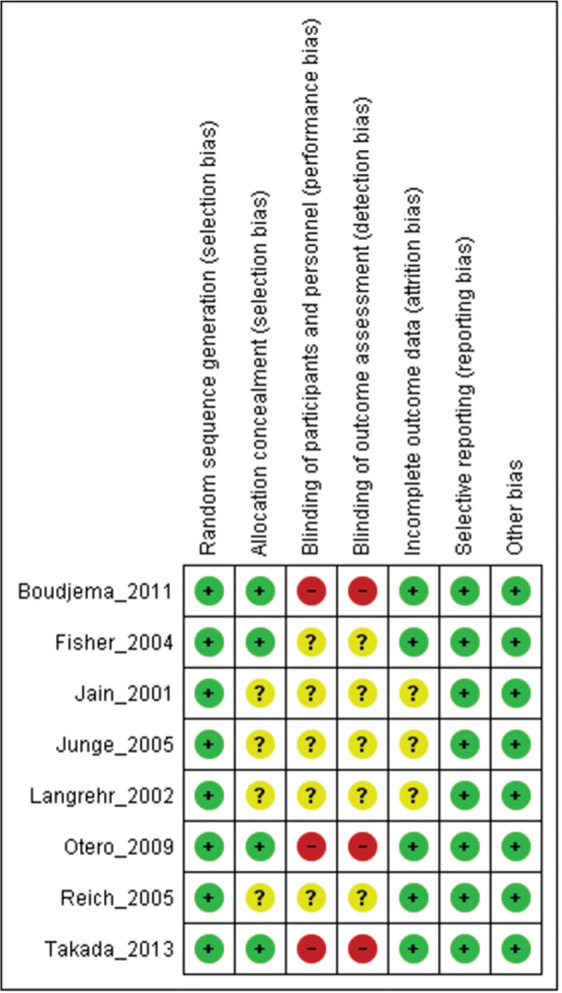
Robins II. Risk of bias evaluation.

**Supplementary File 3:**
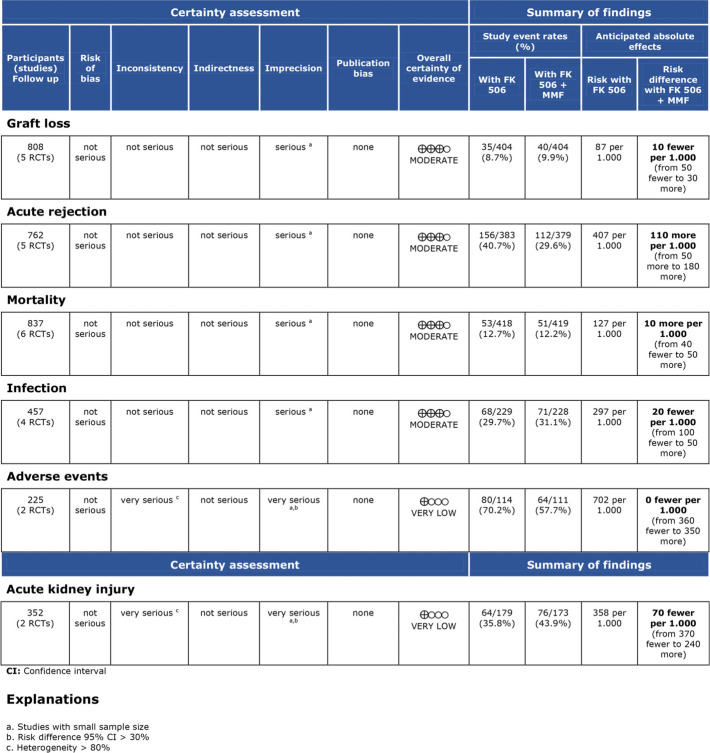
GRADE certainty assessment for comparison FK506 plus MMF vs. FK506 alone. CI: Confidence interval; RCT: randomized clinical trial; MMF: Mycophenolate mofetil; FK506: Tacrolimus; CyA: Cyclosporine; a. Studies with small sample size; b. Risk difference 95% CI>30%; c. Heterogeneity >80%

**Supplementary File 4:**
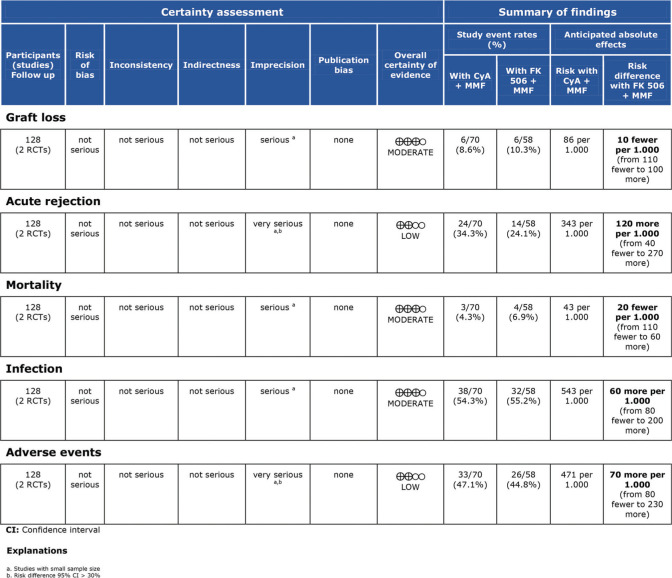
GRADE certainty assessment for comparison FK506 plus MMF vs. CyA plus MMF. CI: Confidence interval; RCT: randomized clinical trial; MMF: Mycophenolate mofetil; FK506: Tacrolimus; CyA: Cyclosporine; a. Studies with small sample size; b. Risk difference 95% CI>30%; c. Heterogeneity >80%.

**Supplementary File 5:**
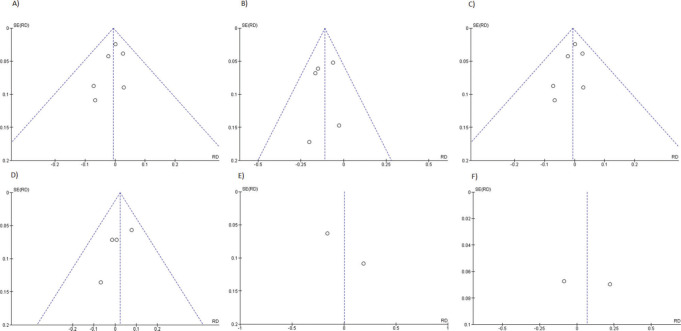
Funnel plots for comparison FK506 plus MMF vs. FK506 alone. A) Graft loss; B) Acute rejection; C) Mortality; D) Infection; E) Adverse events; F) Acute kidney injury. Fixed effect model was used for A, B, and C; Random effect model was used for D and E.

**Supplementary File 6:**
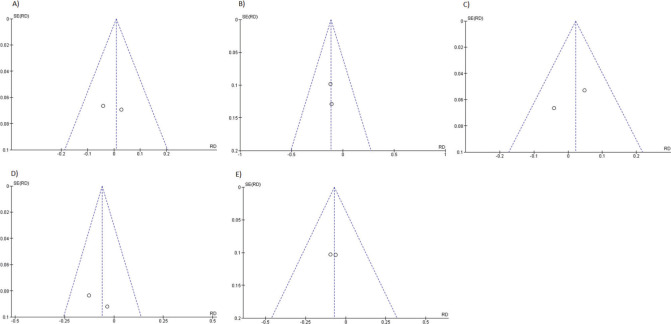
Funnel plots for comparison FK506 plus MMF vs. CyA plus MMF. A) Graft loss; B) Acute rejection; C) Mortality; D) Infection; E) Adverse events. Fixed effect model was used for A, B, C, and D.
